# Versatile supramolecular reactivity of zinc-tetra(4-pyridyl)porphyrin in crystalline solids: Polymeric grids with zinc dichloride and hydrogen-bonded networks with mellitic acid

**DOI:** 10.3762/bjoc.5.77

**Published:** 2009-12-11

**Authors:** Sophia Lipstman, Israel Goldberg

**Affiliations:** 1 School of Chemistry, Sackler Faculty of Exact Sciences, Tel Aviv University, 69978 Ramat Aviv, Tel Aviv, Israel

**Keywords:** coordination polymers, crystal engineering, hydrogen-bonded networks, porphyrin assemblies, supramolecular chemistry

## Abstract

Crystal engineering studies confirm that the zinc-tetra(4-pyridyl)porphyrin building block reveals versatile supramolecular chemistry. In this work, it was found to be reactive in the assembly of both (a) a 2D polymeric array by a unique combination of self-coordination and coordination through external zinc dichloride linkers and (b) an extended heteromolecular hydrogen-bonded network with mellitic acid sustained by multiple connectivity between the component species.

## Introduction

The tetra(4-pyridyl)porphyrin entity in its free-base (TPyP) as well as its metallated (MTPyP) forms has an extraordinarily rich supramolecular chemistry, playing an important role in the construction of diverse polymeric architectures. The TPyPs are readily available [1], and the tendency of the zinc-porphyrin derivative to form polymeric chains by self-coordination between the peripheral pyridyl sites of one molecule and the zinc center of an adjacent species was demonstrated nearly two decades ago [2–3]. Subsequently, it was discovered that MTPyP can not only self-assemble into 1D polymeric arrays, but can also form a robust 3D architecture with molecular sieving features [4]. The facile formation of 3D aggregates, in which the porphyrin units are inter-coordinated via exocyclic metal ion linkers (e.g., tetrahedral Cu^I^), has also been observed [5]. The latter mode of coordination polymerization, in which the peripheral pyridyl sites of different TPyP/MTPyP moieties can be readily bridged by various metal ion connectors due to the high affinity of the pyridyl N-sites to coordinate to transition metal ions, has attracted much attention over the years, leading to the formulation of a large variety of hybrid organic–inorganic 1D ladders and ribbons, 2D nets, and 3D supramolecular constructs (representative refs [6–11]).

Moreover, a new series of MTPyP-based homomolecular coordination polymers has been reported [12–19]. In particular, supramolecular isomerism characterizes the zinc metalloporphyrin compound, which results in a range of coordination aggregates with diverse connectivity patterns [17–19]. The high propensity of the ZnTPyP moiety to exhibit various modes of self-coordination can be attributed to the binding flexibility of the zinc ion, as well as to the multiple potential ligating sites (the four pyridyl substituents) of the square-planar porphyrin framework. Thus, the zinc ion in the porphyrin core can be four-coordinate (to the four pyrrole N-sites without any axial ligation and no option for self-coordination), or, as most frequently encountered, five-coordinate (binding, in addition, one axial ligand), or six-coordinate (in an octahedral environment with two axial ligands on both sides of the porphyrin macrocycle). In the five-coordinate case, the ZnTPyP assemblies are either 1D chain-polymeric or 0D square-oligomeric, whereas in the six-coordinate case, they form either 3D honeycomb architectures or 2D square-grid networks [17–19]. Simultaneous appearance of the two coordination modes in a single homomeric assembly has also been observed, yielding in such a case ladder-type 1D polymeric ribbons [13,15].

The hydrogen-bonding capacity of TPyP and MTPyP in network formation has not been explored until recently. The porphyrin framework is characterized by a square-planar symmetry, bearing laterally diverging pyridyl sites. The latter are available for hydrogen bonding as proton acceptors with complementary components that can act as proton donors. Formulation of extended hydrogen-bonding-sustained networks requires ideally a tetradentate proton donor of similar square-planar symmetry. It has been confirmed that 1,2,4,5-benzenetetracarboxylic acid (B4CA) is perfectly suited for this purpose, as are hydrogen-bonded dimers of 1,3,5-benzenetricarboxylic acid (B3CA) [20–21]. In both cases the TPyP moiety self-assembles with the corresponding acid in appropriate solubilizing environments into 2D heteromolecular grids held together by multiple hydrogen bonding. The same applies to ZnTPyP, when the axial coordination site of the zinc ion is blocked.

**Scheme 1:**
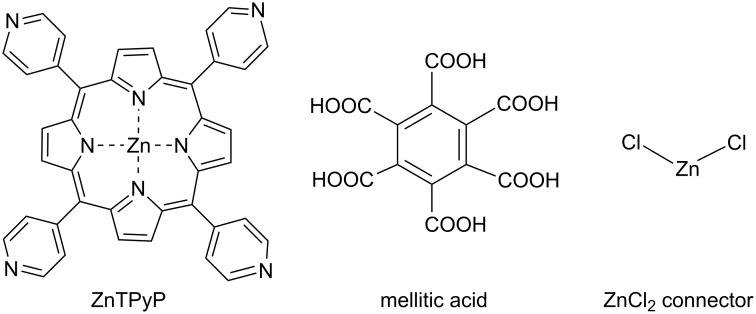
Component building blocks of the supramolecular assembly in **I** and **II**.

In order to expand the library of the available polymeric materials and further explore the different possible modes of self-assembly, we report here on two new ZnTPyP-based structures characterized by either coordination or hydrogen-bonding networking. They represent a coordination polymer ZnTPyP·ZnCl_2_, which crystallizes as a 1,1,2,2-tetrachloroethane (TCE) trisolvate (**I**), and a hydrogen-bonded polymeric network composed of Zn(EtOH)TPyP (where the ethanol solvent occupies and protects the axial coordination site of the zinc ion) and 1,2,3,4,5,6-benzenehexacarboxylic (mellitic) acid (1:1), which crystallizes with one molecule of *o*-dichlorobenzene and three molecules of methanol solvent (**II**). The component building blocks are shown in Scheme 1.

## Results and Discussion

**Figure 1:**
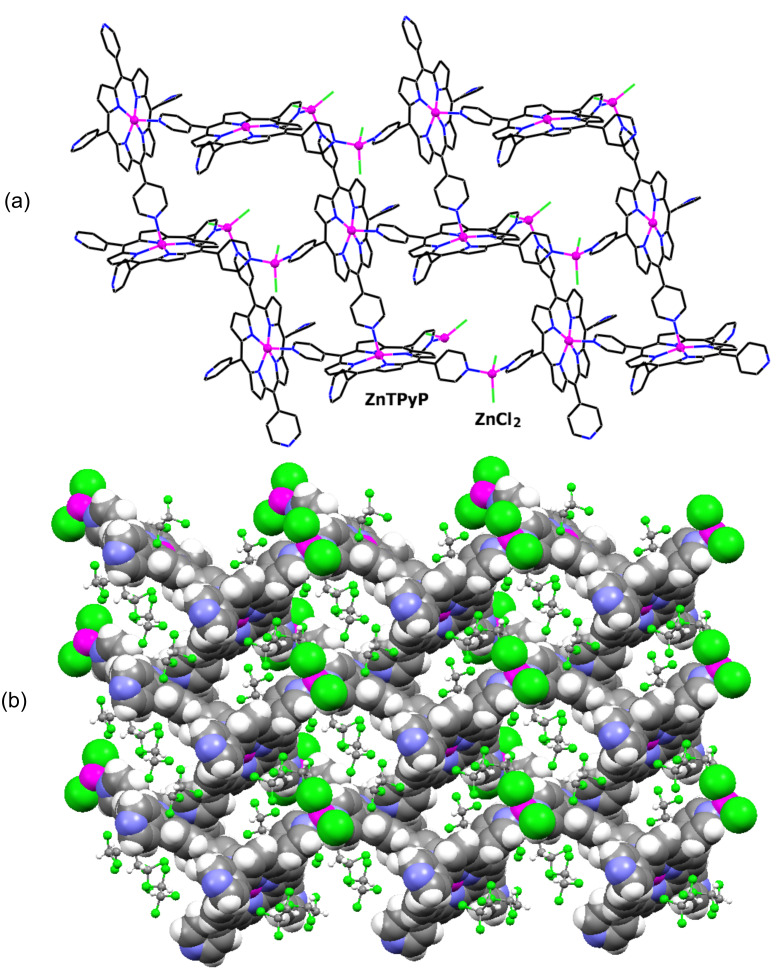
Fragment of the continuous coordination scheme in **I**, forming a corrugated layer that is aligned perpendicular to the *c*-axis of the crystal. (a) Wireframe presentation, with the exception of the zinc ions which are depicted as small spheres, illustrating the connectivity scheme. (b) Space-filling view of the coordination polymer. Note that the ZnCl_2_ bridges and the non-coordinated pyridyl groups point outward from the network. The interporphyrin kinks and voids within the polymeric layer, as well as between neighboring layers, are occupied by the TCE solvent (shown as “ball-and-stick” molecules).

The coordination polymer in structure **I** was obtained by chance, while attempting to network ZnTPyP with different tetracarboxylic acids (see Experimental). It exhibits, however, a uniquely interesting connectivity scheme that combines direct and through-ZnCl_2_ porphyrin-to-porphyrin coordination (Figure 1), a pattern not previously observed.

**Crystal data for I**: C_40_H_24_N_8_·ZnCl_2_·3C_2_H_2_Cl_4_, *M* = 1321.82, monoclinic, space group *P*2_1_/*c*, *a* = 19.1157(2) Å, *b* = 12.9275(2) Å, *c* = 21.8495(2) Å, β = 103.396(1)°, *V* = 5252.5(1) Å^3^, *Z* = 4, *D*_c_ = 1.672 g cm^−3^, μ(Mo Kα) = 1.67 mm^−1^, 38848 reflections measured, 12452 unique (*R*_int_ = 0.045), final *R* = 0.065 for 8437 reflections with *I* > 2σ(*I*) and *R* = 0.099 (*wR* = 0.200) for all data.

The zinc ion in the porphyrin core is five-coordinate with square-pyramidal environment. A given porphyrin unit is involved in two direct coordination bonds to its neighbors. One of the four peripheral pyridyl substituents links to the zinc center of an adjacent species, while the metal ion binds to a pyridyl group of a third porphyrin unit (at Zn–N = 2.150 Å; all the Zn–N_pyrrole_ bond lengths are in the range 2.066–2.075 Å). It is further coordinated to two additional porphyrins with the aid of the tetrahedral zinc dichloride connectors (at Zn–N = 2.040 and 2.046 Å), each bridging between pyridyl groups of two neighboring moieties. The fourth pyridyl group is not involved in intermolecular coordination, and is rotationally disordered in the crystal. Such a four-point per porphyrin binding model, which involves the zinc ion and three of the pyridyl groups, results in the formation of a 2D grid coordination polymer wherein neighboring porphyrins are roughly perpendicular to each other. There is a considerable resemblance between the observed connectivity and that found in the recently reported “paddle-and-wheel”-like square-grid homomolecular coordination polymer of ZnTPyP with a six-coordinate environment around the zinc center [17–18]. The inter-coordinated assembly represents a markedly corrugated layer, which is aligned normal to the *c*-axis of the unit cell. The non-coordinated pyridyl groups and the chloride ions lie above and below the molecular surface of this layer. In the crystals the layers are stacked along the *c*-axis and partly interdigitate into one another. Molecules of the TCE crystallization solvent accommodate the interporphyrin kinks and voids within the polymeric assembly, as well as voids in the interface between adjacent layers. The formation of coordination polymers bridged by exocyclic zinc ions of tetrahedral geometry has also been observed in supramolecular materials based on the tetra(4-carboxyphenyl)porphyrin building blocks [22–23].

Based on previous findings [20–21], the ZnTPyP and mellitic acid components provide excellent building blocks for the construction of heteromolecular networks sustained by cooperative hydrogen bonding. Both have multiple laterally diverging functions, the 4-pyridyl substituents in ZnTPyP acting as proton acceptors and the carboxylic residues in mellitic acid as complementary proton donors. The heteromeric COOH···N_pyridyl_ interaction results in a relatively strong hydrogen bond, which frequently directs supramolecular organization in organic crystals [24]. Preferential hydrogen bonding of ZnTPyP to the mellitic acid, over self-coordination (as in **I**), may occur only if the axial coordination ability of the central zinc ion is blocked. This can be achieved by introducing small zinc-coordinating ligands (e.g., water, MeOH, EtOH, DMF, or DMSO) into the crystallization mixture [20], as in the present case. Ideally, the use of tetracarboxylic ligand of square-planar geometry such as B4CA is best suited to optimize hydrogen-bonding interactions with the complementary tetradentate TPyP moiety [20–21]. When 1,3,5-benzenetricarboxylic acid was used in a similar reaction, it formed hydrogen-bonded dimers first by using one COOH function of each monomer to yield an entity with four free carboxylic groups to bind to the porphyrin.

**Figure 2:**
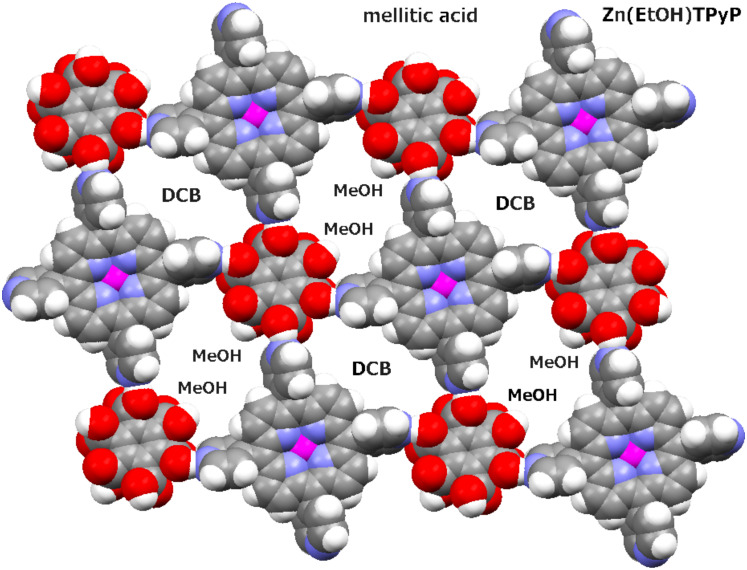
Space-filling representation of the hydrogen-bonded heteromolecular network in **II**. Note that every porphyrin unit is in direct hydrogen-bonding contact through its pyridyl groups with four molecules of mellitic acid and vice versa. Two of the carboxylic groups of the latter point into the intralayer voids. They are solvated in the crystal by molecules of the methanol solvent (MeOH) that occupy the adjacent voids, the alternating voids being occupied by the *o*-dichlorobenzene (DCB) solvent. These flat layers are aligned parallel to the (110) plane of the crystal. In this figure, at every porphyrin site the EtOH axial ligand is connected to the central zinc ion from below, preventing self-coordination of the ZnTPyP units (as in **I**).

Not surprisingly, therefore, when the mellitic acid was used in this study, two of its carboxylic acid functions (at positions 3 and 6 of the central benzene ring) did not interact with Zn(EtOH)TPyP, thus mimicking effectively the functionality of B4CA. The supramolecular assembly that formed in this case is depicted in Figure 2.

**Crystal data for II**: C_40_H_24_N_8_Zn·C_2_H_5_OH·C_12_H_6_O_12_ (crystallization solvent, *X* = C_6_H_4_Cl_2_·3CH_3_OH, excluded due to disorder), *M* = 1070.28, triclinic, space group *P*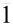, *a* = 11.8660(3) Å, *b* = 13.8803(4) Å, *c* = 20.4843(5) Å, α = 78.237(2)°, β = 78.185(2)°, γ = 84.288(1)°, *V* = 3227.1(2) Å^3^, *Z* = 2, *D*_c_ = 1.101 g cm^−3^ and μ(Mo Kα) = 0.44 mm^−1^ (*X* excluded), 35843 reflections measured, 15151 unique (*R*_int_ = 0.051), final *R* = 0.060 for 9766 reflections with *I* > 2σ(*I*) and *R* = 0.092 (*wR* = 0.167) for all data. Molecules of the crystallization solvent are included in a severely disordered manner within the interstitial voids of the crystal lattice (the solvent-accessible voids amount to 35.3% of the crystal volume) and cannot be modeled reliably by discrete atoms. Conventional least-squares refinement of the complete structural model resulted in *R*1 = 0.10. In the final calculations the contribution of the disordered solvent was subtracted from the diffraction data by the Squeeze procedure [25], a common practice in similar situations. The least-squares refinement converged smoothly to a lower *R*-value, allowing a precise determination of the hydrogen-bonded network.

Compound **II** is characterized by a 1:1 stoichiometry of the Zn(EtOH)TPyP and mellitic acid components. Every porphyrin is efficiently hydrogen bonded to four adjacent acids, and every acid is hydrogen bonded to four different porphyrins. This connectivity scheme results in a fascinating supramolecular grid sustained by such cooperative COOH···N_py_ hydrogen bonding (at N···O within 2.56–2.62 Å). The layered array thus formed has an open structure, with voids of two types lined by the two pairs of the networking components and encircled by four hydrogen bonds (Figure 2). The two “excess” carboxylic functions of the mellitic acid are solvated by the methanol solvent that protrudes into 50% of the intralayer voids. The other voids within the layered array are filled by DCB. Such neighboring layers are related to one another by inversion in an offset manner. Within the inversion-related layers the axial EtOH ligands of one layer penetrate into the voids of another layer. The concave surfaces of the five-coordinate porphyrin moieties are located on the outside of the paired layers, inducing further incorporation of crystallization solvent (MeOH) to fill this interfacial space between the paired layers, which stack effectively along the [110] axis of the crystal.

## Conclusion

This study confirms the high versatility of the ZnTPyP moiety as an effective building block in the formulation of supramolecular grids. The square-planar ZnTPyP framework has diverse and multidentate binding capacities. It can self-coordinate directly via the Zn-pyridyl bonds to form 2D and 3D polymeric arrays [4,18–19]. Then, it can form supramolecular assemblies of varying dimensionality with the aid of exocyclic metal ion linkers capable of coordinating simultaneously to several neighboring ZnTPyP units [5–11]. The ZnTPyP can adopt in the above constructs either five-coordinate or six-coordinate geometries, which affects the architecture of the resulting assembly. Formation of coordination networks by combining direct porphyrin–porphyrin coordination and coordination through an external linker (as in **I**) has been demonstrated here for the first time. It has further been demonstrated that TPyP and ZnTPyP scaffolds may be used also for the formulation of supramolecular assemblies sustained by cooperative hydrogen bonding with the pyridyl substituents as excellent proton acceptors for compatible proton donating species [20–21]. In particular, the strong COOH···N_py_ interaction [24] can be harnessed to this end by reacting the tetrapyridylporphyrin with a polycarboxylic acid entity, and thus inducing cooperative hydrogen-bonding interactions in four different directions. The observed structure of compound **II** provides an attractive example of designed formulation of such heteromolecular networks. The observed modes of self-assembly are of further significance to studies of surface-based crystallizations of monolayer and multilayer hydrogen-bonded networks and metal–organic frameworks on various substrates [26–27], in the context of the design of novel molecular devices.

## Experimental

The ZnTPyP, 1,4,5,8-naphthalenetetracarboxylic acid (NTCA), 1,2,3,4,5,6-benzenehexacarboxylic (mellitic) acid, 3,4,9,10-perylenetetracarboxylic acid (PTCA), as well as common laboratory solvents were procured commercially, and used without further purification. The porphyrin was treated with different acids under diverse experimental conditions in an attempt to synthesize heteromeric hydrogen-bonding networks of the interacting components. The coordination polymeric compound (**I**) was first obtained when a methanol solution of PTCA (in which ZnTPyP is sparingly soluble) was carefully layered at room temperature over a solution of ZnTPyP (0.015 mmol) in a 1:1 mixture of TCE and methanol (10 ml). Crystals appeared in the bottom solution after a few days. The same crystalline compound was obtained under reflux conditions when 0.05 mmol of ZnTPyP was reacted with 0.05 mmol of NTCA in a 1:1:1:1 solvent mixture of TCE, *o*-chlorophenol/*o*-dichlorobenzene, ethanol, and *N*,*N*-dimethylformamide (DMF). The resulting solution was refluxed for 16 h, and then cooled to room temperature and left for crystallization. X-ray quality crystals were obtained after four days. It appeared in both cases that ZnCl_2_ was formed in situ under the acidic conditions by extracting some of the zinc ions from the metalloporphyrin. The porphyrin-mellitic acid hydrogen-bonded compound (**II**) was obtained when a methanol solution (10 ml) of mellitic acid (0.055 mmol) was carefully layered over a solution of 0.010 mmol of ZnTPyP dissolved in a 1:1 mixture (10 ml) of ethanol and *o*-dichlorobenzene. Sizeable crystals appeared after three days. The uniformity of the formed crystalline materials was confirmed in each case by repeated measurements of the unit cell dimensions from several randomly chosen single crystals.

The diffraction measurements were carried out on a Nonius KappaCCD diffractometer, using graphite monochromated Mo Kα radiation (λ = 0.7107 Å). The crystalline samples of the analyzed compounds were covered with a thin layer of light oil and freeze-cooled to ca. 110 K in order to minimize solvent escape, structural disorder, and thermal motion effects, and increase the precision of the results. The structures were solved by direct methods (SIR-97) and refined by full-matrix least-squares on *F*^2^ (SHELXL-97). Intensity data were corrected for absorption effects. All non-hydrogen atoms (except of those of the disordered pyridyl group and TCE solvent in **I** and the disordered solvent in **II**) were refined anisotropically. The hydrogens were either found in difference Fourier maps or located in idealized positions, and were refined using a riding model with fixed thermal parameters [*U_ij_* = 1.2 or 1.5 *U_ij_* (eq.) for the atom to which they are bonded]. No phase transitions of the two crystalline compounds were detected between room temperature and 110 K. The two polymeric structure types contain sizeable voids, which are accommodated by molecules of crystallization solvent (three molecules of TCE in **I**, and one moiety of *o*-dichlorobenzene and three molecules of methanol in **II**). In **II**, the solvent species could be clearly identified in the electron-density maps but they were found to be severely disordered in the lattice and could not be reliably modeled by discrete atoms. Correspondingly, their contribution to the diffraction pattern was subtracted by the Squeeze procedure (commonly used in similar situations) [25], allowing smooth convergence of the crystallographic refinement and precise description of the hydrogen-bonded framework.

## Supporting Information

Supporting information features X-ray data for compounds **I** and **II**.

File 1 X-ray data for compound **I**.

File 2 X-ray data for compound **II**.
